# Lipoprotein(a) and cardiovascular disease

**DOI:** 10.1042/BCJ20240037

**Published:** 2024-09-20

**Authors:** Michael B. Boffa, Marlys L. Koschinsky

**Affiliations:** 1 Department of Biochemistry, University of Western Ontario, London, Ontario, Canada; 2 Robarts Research Institute, University of Western Ontario, London, Ontario, Canada; 3 Department of Physiology and Pharmacology, University of Western Ontario, London, Ontario, Canada

**Keywords:** apolipoprotein(a), atherosclerotic cardiovascular disease, calcific aortic valve disease, lipoprotein metabolism, lipoprotein(a), thrombosis

## Abstract

Elevated plasma levels of lipoprotein(a) (Lp(a)) are a prevalent, independent, and causal risk factor for atherosclerotic cardiovascular disease and calcific aortic valve disease. Lp(a) consists of a lipoprotein particle resembling low density lipoprotein and the covalently-attached glycoprotein apolipoprotein(a) (apo(a)). Novel therapeutics that specifically and potently lower Lp(a) levels are currently in advanced stages of clinical development, including in large, phase 3 cardiovascular outcomes trials. However, fundamental unanswered questions remain concerning some key aspects of Lp(a) biosynthesis and catabolism as well as the true pathogenic mechanisms of the particle. In this review, we describe the salient biochemical features of Lp(a) and apo(a) and how they underlie the disease-causing potential of Lp(a), the factors that determine plasma Lp(a) concentrations, and the mechanism of action of Lp(a)-lowering drugs.

## Introduction

Lipoprotein(a) (Lp(a)) was first reported in 1963 as an antigenic variant of low-density lipoprotein (LDL), and was detectable in plasma from approximately one third of a Northern European population [1]. While numerous case-control studies dating back to the 1970s identified elevated Lp(a) (>30 mg/dl; >75 nmol/L) in coronary heart disease (CHD) patients, the contribution of Lp(a) to cardiovascular disease (CVD) risk remained unclear until many decades later. In three landmark studies published in 2009, Lp(a) was shown to be an independent and casual risk factor for atherosclerotic cardiovascular disease (ASCVD) [2–4], and in 2013, the importance of Lp(a) as a potent risk factor for the initiation and progression of aortic valve stenosis was reported [5].

**Figure 1.**
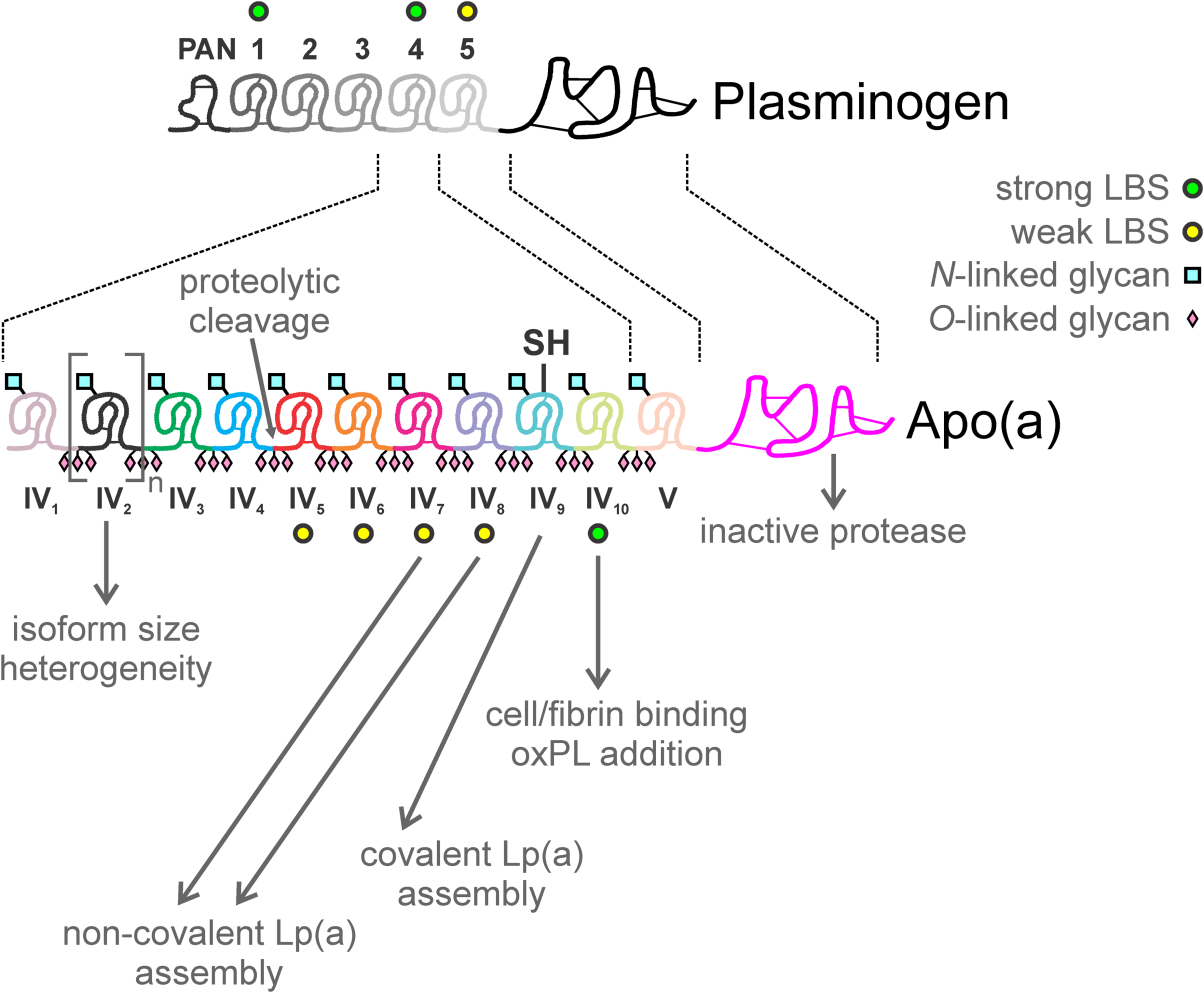
Kringle organization and functional domains of apo(a). Apo(a) consists of multiply-repeated domains homologous to plasminogen kringle 4, followed by a single kringle 5-like domain and an inactive protease-like domain. Kringle UIV type 2 in apo(a) (KIV2) is present in between 3 and over 40 copies and underlies Lp(a) isoform heterogeneity in the population. Some apo(a) KIV types contains weak or strong lysine binding sites (LBS) that play roles in Lp(a) assembly, binding to Lp(a) to substrates, and addition of oxidized phospholipid (oxPL). Apo(a) KIV9 contains the single unpaired cysteine that mediates disulfide bonding to apoB100 in the Lp(a) particle. Also indicated is the presence of *N*- and *O*-linked glycosylation in each kringle and in the interkringle regions, respectively, and the site of proteolytic cleavage by elastase or MMP-12.

Lp(a) contains an apoB-100-associated lipid component that is similar (but not identical) to LDL [6,7]. Importantly, a hallmark feature of Lp(a) is the presence of apolipoprotein(a) (apo(a)), which is unique amongst apolipoproteins both in its structure and its biochemical properties. Characterization of a human liver-derived apo(a) cDNA in 1987 revealed that apo(a) is very similar to the fibrinolytic proenzyme plasminogen [8] (Figure 1). In this regard, apo(a) contains numerous kringle domains that closely resemble several of those found in plasminogen. Kringle domains are structural motifs primarily found in proteins involved in the coagulation and fibrinolytic pathways. Each kringle domain contains three invariant disulfide bonds. Kringles are not present in any other apolipoproteins, and evidence continues to accrue that the kringles in apo(a) impart the majority of pathophysiological properties that are associated with Lp(a). The unexpected finding of a high level of sequence similarity between apo(a) and plasminogen led to speculation that apo(a) may interfere with normal functions of plasminogen in the breakdown of fibrin clots. Therefore, it was hypothesized that Lp(a) could provide a unique link between atherosclerosis (due to the apoB-containing lipid component of Lp(a)) and thrombosis (attributable to the apo(a) component of Lp(a)). This potential duality of function has sparked many studies and debates on the role of Lp(a) in cardiovascular pathology.

Plasma levels of Lp(a) vary widely in the global population, ranging from <1 mg/dl (2.5 nmol/L) to >250 mg/dl (625 nmol/l). Cardiovascular risk thresholds have been defined as >30–50 mg/dl, (75–125 nmol/l) with over 20% of the global population having levels of Lp(a) in excess of 50 mg/dl (125 nmol/l) [9]. Unlike LDL, a large component (more than 90%) of Lp(a) levels is genetically determined, and plasma Lp(a) levels reflect a co-dominant inheritance pattern [10]. The strong genetic determination of Lp(a) levels cannot be attributed solely to *LPA* isoform size variability. Other genetic variants in *LPA* have been identified — including nonsense mutations and splice site mutations — that help explain vast differences in Lp(a) levels in individuals with identical isoform sizes [11]. Non-genetic factors (diet and hormones, for example) can also affect Lp(a) levels although the contributions are relatively modest [12,13].

## Structure-function relationships in apo(a)

### Structure of Lp(a) and apo(a)

Lp(a) is a spherical particle of ∼250 angstroms in diameter that floats in the higher density range of LDL particles [14]. It has been shown that apo(a) and apoB-100 exist in a 1:1 molar ratio in the Lp(a) particle [15]. The apo(a) component of Lp(a) is heavily glycosylated (∼28% carbohydrate by weight) with a high content of sialic acid [6].

The hallmark of the apo(a) protein is the presence of multiply-repeated kringle IV (KIV) domains that are very similar to plasminogen kringle 4 (75–85% sequence identity); these are followed by a single copy of sequences that closely resemble the plasminogen kringle 5 (KV) and protease domains of plasminogen (91 and 84% sequence identity, respectively) (Figure 1) [8]. Kringles are structural motifs containing three invariant disulfide bonds forming a compact tri-loop structure generally lacking any discernable regular secondary structure [16]. Typically, kringles are present in proteins involved in the fibrinolytic (e.g. tissue-type plasminogen activator (tPA) and plasminogen) and coagulation (e.g. prothrombin) pathways. Many kringles are involved in ligand interaction through a binding cleft characterized by an anionic center and a cationic center separated by a hydrophobic trough. Several of the five kringle domains in plasminogen contain specificity for lysine binding; the strength of the interaction is K1 > K4 > K5. In apo(a), the KV and several KIV domains each contain binding clefts which differ in their specificity for lysine and other amino acids compared with plasminogen [17–21].

Early studies demonstrated heterogeneity in apo(a) sizes from Lp(a) isolated from different individuals; it was observed that plasma Lp(a) isolates varied in density, with smaller-sized apo(a) species corresponding to less dense Lp(a) particles. In 1992 the Koschinsky group reported the core kringle organization of apo(a) using a PCR-based approach [22]. In this hallmark study, it was determined that apo(a) contains 10 kringle invariant plasminogen KIV-like domains (KIV1–KIV10) that are highly similar to each other but contain some key differences in amino acid sequence; the KIV domains are followed by a single copy of each of the KV and protease-like domains. The apo(a) KIV2 domain is present in different numbers of identically-repeated copies in human Lp(a), from 3 to >30 [23]. This corresponds to apo(a) isoform sizes ranging from <200 to >800 kDa that represent copy-number variation of the KIV2-encoding sequence in the *LPA* gene. This forms the molecular basis of plasma Lp(a) isoform size heterogeneity which is a hallmark of Lp(a).

The protease-like domain of apo(a) was shown to lack proteolytic activity despite the retention of the canonical catalytic triad residues found in serine proteases [24]. Modeling of the protease domain showed that an 8 amino acid deletion in the protease-like domain of apo(a) causes collapse of the active site, with a resulting lack of enzymatic activity. Additionally, the tPA cleavage site in plasminogen (arginine) that is required for plasminogen activation to plasmin is replaced by a serine residue in apo(a). Studies have shown that apo(a) contains no inherent proteolytic activity which is reflective of these differences [24]. The lack of catalytic activity in apo(a) analogous to that of plasmin(ogen) has led to speculation that apo(a) can compete with the proteolytic function of plasmin in cleaving fibrinogen to fibrin. This, in turn, has led to speculation that Lp(a) possess antifibrinolytic properties, which contribute to its pathogenic effects in the vasculature.

### Lysine-binding properties of apo(a) kringles

**Figure 2.**
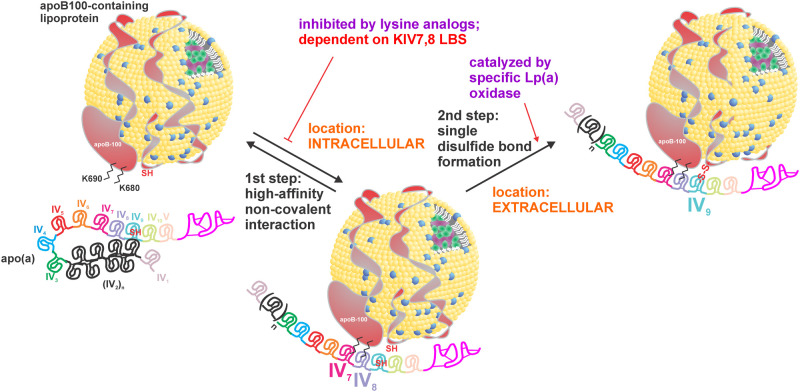
Assembly of Lp(a). Within the hepatocyte, apo(a) KIV7 and 8 form an initial non-covalent interaction with specific lysine residues in the amino-terminal domain of apoB100. Covalent bond formation occurs extracellularly and is catalyzed by a specific oxidase enzyme. Also depicted is the ligand-induced conformation change in apo(a) that accelerates covalent Lp(a) assembly [26].

Extensive structure/function analyses have been carried out on the kringle domains of apo(a) through the use of recombinant protein engineering and expression of apo(a). Expression of a 17-kringle containing isoform of apo(a) was reported in 1991 [25]. Following that, many recombinant apo(a) proteins have been generated to study the roles of specific kringle domains. We know, for example, that apo(a) KIV types 5–8 (KIV5–8) each contain a weak lysine-binding site (LBS), several of which are important in the biogenesis of the Lp(a) particle (Figures 1 and 2; see section ‘Assembly of Lp(a) particles’, below) and that the KIV type 10 (KIV10) domain in apo(a) has a LBS of comparable affinity to that in plasminogen KIV [27]. It was also reported that the single covalent bond that links apo(a) to apoB-100 in the Lp(a) particle is located in apo(a) KIV type 9 (KIV9) [28]; this is the only unpaired cysteine residue present in the apo(a) molecule. The identity of the corresponding cysteine in human apoB-100 is Cys4326 which is located in the C-terminal region of the molecule [29].

The crystal structures of apo(a) KIV2, KIV7 and KIV10, as well as KV have been solved [17,19–21]. While the overall structure of full-length apo(a) has not been reported, a model of the structure of three KIV domains (KIV types 8, 9 and 10) has been published [30]. Interestingly, the trigonal arrangement of these three kringles in the resulting model resembles the asymmetric unit of three KIV_2_ molecules observed in the crystal structure of KIV2 [21].

The structure of the LBS in kringle domains generally consists of a hydrophobic trough on the surface of the kringle, with clusters of anionic and cationic residues present on either end. The apo(a) KIV_10_ LBS consists of seven essential residues comprising the hydrophobic trough (Trp^60^, Phe^62^ and Trp^70^; numbering is relative to the first Cys in the kringle), the cationic center (Asp^54^ and Asp^56^) and the anionic center (Arg^35^ and Arg^69^). Other apo(a) KIV types contain substitutions in one or more of these residues that weaken or eliminate lysine binding. These include substitutions at position 56 which is cationic (Asp or Glu) in the lysine binding kringles, but neutral (Val, Gly) in the others. The X-ray crystal structure of KIV_7_ revealed a smaller LBS compared with KIV_10_, resulting from mutations of the critical residues Asp^56^ and Tyr^62^ in the LBS to Glu and Phe, respectively [17]. The Asp^56^ à Glu substitution is also present in KIV_5-8_ each of which bind lysine analogues more weakly compared with apo(a) KIV_10_ or plasminogen kringle 4. These differences in affinity to lysine may underlie (or at least be compatible with) the distinct functional roles of the weak and strong LBS in apo(a) in Lp(a) assembly and binding to lysine-containing substrates, respectively (see below) [31].

The kringle V domain in apo(a) is very similar (91% amino acid sequence identity) to the corresponding domain in plasminogen and has comparable weak lysine binding properties. It is interesting to note that *LPA* in many Old World monkeys lacks the KV domain in the apo(a) protein. There may be implications for apo(a) structure and function based on whether or not this domain is present. For example, it has been reported that in baboon apo(a), which lacks the KV domain, the LBS in KIV10 is non-functional despite the presence of the residues identified as essential for the binding of lysine [32].

### Post-translational modifications of apo(a)

#### Glycosylation

Analysis of the structures of the *N*- and *O*-linked glycans in apo(a) has been performed using exoglycosidase digestion and mass spectroscopy analysis [33]. it was reported that two major *N*-glycans account for ∼17% of the oligosaccharide structures. These *N*-glyans are complex biantennary structures that exist in either a mono-or disialylated state. The *O*-linked glycans, representing the bulk of the carbohydrate modification in apo(a), were reported to be primarily monosialylated core type I structures (NeuNAcα2-3Galβ1-3GalNAc), with smaller amounts of disialylated and non-sialylated structures also present.

The KIV domains in apo(a) are separated by interkringle sequences that are between 30 and 36 amino acids in length. Interestingly, the plasminogen interkringle sequences are significantly shorter. Each of the interkringle regions in apo(a) contains six sites for *O*-linked glycan attachment, with at least one *N*-linked glycosylation site present in each KIV domain [8]. It is interesting to note that apo(a) is very highly glycosylated compared with plasminogen: the latter contains only one *N*-linked and one *O*-linked glycosylation site. The significance of the extensive glycosylation of apo(a) remains unclear.

#### Proteolysis

Proteolytic fragments of apo(a) have been identified in human plasma and urine corresponding to the N-terminal region of apo(a) (KIV_1–4_); these fragments are not attached to apoB-100 [34]. Several studies have reported the sensitivity of plasma Lp(a) to elastase cleavage: the cleavage site has been identified as the Ile-Leu bond in the interkringle sequence between apo(a) KIV_4_ and KIV_5_ [35]; there is also a macrophage metalloelastase (MMP-12) cleavage site (Asn-Val bond) immediately upstream of the elastase cleavage site [36]. The origin and significance of the fragments of apo(a) remain unclear: it is possible that they arise in the vessel wall at the site of atherosclerotic lesions where Lp(a) accumulates, and can diffuse back into the plasma compartment. Interestingly, the interkringle region between apo(a) KIV_4_ and KIV_5_ is deficient in *O*-linked glycans due to amino acid substitutions in serine and/or threonine residues; this renders the sequence more susceptible to proteolytic cleavage compared other interkringle sequences in apo(a). Although the significance of the proteolytic cleavage remains unknown, is tempting to speculate that the role of the extensive *O*-linked glycosylation modification in the interkringle regions serve to protect apo(a) from proteolytic degradation.

#### Oxidized phospholipid addition to Lp(a) and apo(a)

Studies have firmly established that Lp(a) is the preferential carrier of oxidized phospholipids in human circulation [37]. The majority ( ∼85%) of oxPL in plasma is carried by Lp(a), and of this, 50% is associated with the apo(a) component of the particle [37]. These studies used E06, a murine immunodominant IgM antibody isolated from apoE knockout mice, that binds to the phosphocholine head group of oxidized (but not native) oxPL derived from phosphatidylcholine (PC) [38]. E06 recognizes oxPL on an equimolar basis when present either as a PC salt, or as PC on oxPL such as 1-palmitoyl-2-(5-oxovaleroyl)-sn-glycero-3-phosphocholine (POVPC), or attached to a variety of different peptides regardless of amino acid sequence [39]. These oxidized species are highly proinflammatory, and have been linked to early events in atherogenesis through their ability to promote inflammatory cascades in the vessel wall [40]. OxPL are associated with plaque vulnerability and destabilization, and can accumulate at high levels in lesions relative to their plasma concentrations.

The covalent addition of oxPL to proteins can occur through Schiff base formation with the ε-amino group of lysine residues, or by Michael addition to cysteine, histidine, or arginine residues [41]. Studies using a wide variety of r-apo(a) variants pinpointed a role for KIV10 and, specifically, the strong LBS (sLBS) in this kringle in mediating E06-reactive covalent oxPL addition (Figure 1) [42]. In transgenic mice expressing a truncated form of human apo(a) and human apoB-100 (i.e. Lp(a)-transgenic mice), oxPL was present on Lp(a) containing wild-type KIV10, but not the KIV10 sLBS mutant [42]. A role for the KIV10 sLBS in oxPL modification of apo(a) is also implied by the fact that non-human primates lack a functional KIV10 LBS as well as E06-reactive oxPL on their Lp(a). However, whether the sLBS plays a direct or indirect role in oxPL addition to apo(a) is unclear. Although the E06-detectable oxPL corresponds to oxidized phosphatidylcholine, the identity of the presumably oxidized sn-2 fatty acyl chain and the nature of its covalent linkage to apo(a) remain to be determined. Additionally, whether the OxPL modification of apo(a) occurs intra-or extracellularly is not known. Finally, little is known about the nature of the oxPL that are not covalently associated with apo(a), and are therefore extractable from Lp(a) using organic solvents [37]. Moreover, a recent lipidomic study showed that Lp(a) is enriched in (non-oxidized) diacylglycerols and lysophosphatidic acid, and that these species are able to evoke pro-inflammatory responses in monocytes [43].

### Comparative evolution of the gene encoding apo(a)

The *LPA* gene, located on the long arm of chromosome (6q21) very likely arose from a gene duplication of the *PLG* gene: the two genes are in a head-to-head arrangement on the distal end of the long arm of chromosome 6 (6q25.3-q26). Interestingly, the *LPA* gene is absent in all species except for Old World monkeys, great apes and humans. This suggests that the gene duplication event giving rise to human *LPA* occurred <6 million years ago, subsequent to the divergence of Old and New World monkeys [44]. Like humans, the Old World monkeys and apes all lack functional protease domains owing to amino acid substitutions that inactivate this domain in a variety of ways in different species [32,45,46]. Additionally, none of the Lp(a) species in Old World Monkeys and apes contain a functional LBS in their respective KIV10 domains: this results from a combination of amino acid substitutions in the strong LBS and/or the lack of a sequence encoding apo(a) kringle V [32]. All Old World monkey and ape species exhibit apo(a) isoform size variability arising from different numbers of identically repeated copies of the apo(a) KIV2 domain as seen in human Lp(a).

A DNA sequence was cloned from hedgehogs (primitive insectivores) that contains multiple repeats of a sequence that is similar to plasminogen kringle 3, but lacks sequences corresponding to the plasminogen K4, K5 and protease-like domains of apo(a) [47]. Although this also likely arose from a gene duplication of the K3 encoding sequence in *PLG*, the similarity to human apo(a), apart from the presence of a multiply-repeated plasminogen kringle domain, is relatively low. Interestingly, however, there is a free cysteine in hedgehog apo(a) that allows it to form a covalent bond with apoB-100 in the context of hedgehog plasma Lp(a) particles [47]. Apart from that found in hedgehogs, Lp(a)-like species do not appear in evolution prior to the second plasminogen gene duplication event that gave rise to Lp(a) in monkeys, apes and humans. This suggests that the *LPA* gene was lost from the genome early in mammalian evolution.

## The biosynthesis and catabolism of Lp(a)

**Figure 3.**
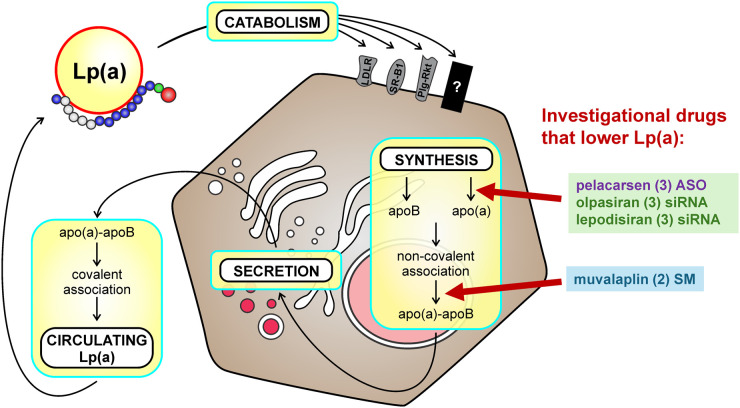
Biosynthesis and catabolism of Lp(a). Novel therapeutics that specifically lower Lp(a) act by destabilizing the *LPA* mRNA that encodes apo(a) or by blocking the initial non-covalent interaction between apo(a) and apoB. After secretion of the non-covalent apo(a)-apoB complex, covalent Lp(a) is formed, and the circulating particle is taken up by the liver by one or more receptors. Candidates for which there is the most evidence are LDL receptor (LDLR), scavenger receptor-B1 (SR-B1) and the plasminogen receptor Plg-R_KT_. The current phase of clinical trial development is listed n parentheses after the drug name. ASO, antisense oligonucleotide; siRNA: small interferening RNA; SM, small molecule.

It was reported in the 1990s that levels of Lp(a) are determined primarily at the level of production rather by the rate of removal from the circulation. These studies were performed in humans using injections of radiolabelled Lp(a) [48,49]. However, there are many steps that comprise production of Lp(a) at which regulation may occur. These include evidence for transcriptional regulation, regulation of secretion, and the process of Lp(a) particle summary, which are summarized below (Figure 3). Understudied areas include possible regulation of *LPA* transcript stability and translation efficiency. With respect to the role of catabolism in determining plasma Lp(a), changes in Lp(a) removal from circulation may be altered in response to disease states including renal disease.

### Transcriptional regulation of *LPA*

The *LPA* gene, although expressed constitutively, is responsive to transcriptional regulation, with regulatory elements located throughout the intergenic region between *PLG* and *LPA*. Regulation of *LPA* expression by estrogen through estrogen receptor binding to a negative enhancer estrogen response element in the *LPA* promoter has been reported [50]. Estrogen therefore decreases *LPA* expression which is reflected in the increase in Lp(a) (up to 27%) that occurs post-menopause [51]. Consistent with this, Lp(a) levels also decrease in response to hormone replacement therapy [52–54]. The FXR (Farnesoid X receptor) ligand binds to a negative control element in the *LPA* promoter and decreases *LPA* expression [55]. Additionally, Fibroblast growth Factor-19 (FGF-19) binding to hepatocytes results in ELK-1 translocation to the nucleus that in turn decreases *LPA* expression [56]. However, neither of these agents have been shown to decrease Lp(a) levels in humans in a clinical context. Activators of *LPA* expression have also been identified: both NHF1A and IL-6 induce *LPA* transcription [57], and an IL-6 receptor blocker (tocilizumab) lowers *LPA* in response to IL-6 [58].

Interestingly, *LPA* can be up-regulated by proinflammatory mediators such as IL-6 as mentioned above, and can also itself serve to increase the expression of inflammatory factors such as IL-8 [59]. This has led to speculation that elevated Lp(a) levels may pose a major risk to patients with COVID-19 in response to the ensuing ‘cytokine storm’ associated with this virus [60].

### Secretion of apo(a) from hepatoctyes

Plasma Lp(a) contains apo(a) and apoB-100, both of which are produced in hepatocytes. Given the lack of detectable apo(a) in both primary and cultured hepatocytes, much of our understanding of apo(a) synthesis and secretion has been obtained using either immortalized cells lines or primary hepatocytes derived from baboon liver.

The baboon liver hepatocyte model has provided critical information regarding the posttranslational control of apo(a) secretion [61]. Apo(a) is synthesized as a precursor containing high-mannose *N*-linked glycans that have a prolonged residence time in the ER prior to maturation and secretion. Maturation occurs in the Golgi and involves modification of *N*-linked glycans and the addition of *O*-linked carbohydrate side chains including sialic acid [62].

White et al. [63,64] showed that the strong inverse correlation between plasma Lp(a) levels and apo(a) isoform sizes could be explained in large part by the increased ER retention time for larger apo(a) species. This reflects greater complexity in folding requirements of larger isoform sizes, resulting in increased degradation through the ERAD pathway in which misfolded species are translocated across the ER membrane followed by ubiquitination. Whether or not other processes regulate apo(a) presecretory degradation remains unclear since it has been reported that only a portion of apo(a) is targeted for proteasomal degradation [65].

Using the same model, the White group showed that apo(a) folding begins co-translationally during import into the ER lumen. The nascent protein progresses through a series of intermediately-folded species during which multiple chaperones such as PDI, calnexin and calreticulin play key roles in ensuring that protein folding is complete [65,66]. It is important to note that *N*-linked glycan addition and trimming in the ER is required for apo(a) secretion, and mediates critical interactions with calnexin and calreticulin that ensure proper folding of nascent apo(a) prior to its export from the ER. Notably, White et al. [63] showed that all apo(a) isoforms folded at the same rate regardless of their size. The size-sensitive process controlling the rate of ER exit remains unknown, but is dependent on the trimming of *N*-linked glucose.

### Assembly of Lp(a) particles

It has been firmly established that Lp(a) particle assembly proceeds as a two-step process: the first step involves non-covalent interactions that occur between apo(a) and apoB-100 prior to the second step of assembly that involves the formation of a single disulfide bond between apo(a) and apoB-100 (Figure 2) [31]. The non-covalent step of Lp(a) assembly involves lysine-dependent interactions between weak LBSs present in each of apo(a) KIV types 7 and 8, with lysine residues present in the amino-terminal globular domain of apoB-100 [67]. As such, Lp(a) assembly can be effectively inhibited by lysine analogues *in vitro* through interference with the first step of the process. The second step of Lp(a) assembly involves formation of a covalent bond between the free cysteine in apo(a) KIV9 and the unpaired cysteine at amino acid position 4326 in human apoB-100.

An active field of Lp(a) assembly has involved determination of whether the process occurs intracellularly or extracellularly. A number of investigators have reported the inability to detect intracellular covalent Lp(a) particles using cultured hepatocyte cell models and human liver tissue [28,62,68]. White et al. [62,68] work in cultured baboon hepatocytes suggested that assembly of Lp(a) occurs extracellularly, on the hepatocyte cell surface. Becker et al. [69] showed that an enzyme with oxidase activity present in cultured hepatocyte cell medium is responsible for extracellular covalent bond formation between apo(a) and apoB-100. They also demonstrated that covalent bond formation requires opening of the apo(a) structure using a lysine analogue [26]. This presents an intriguing possibility where secreted apo(a) may bind to a receptor(s) on the cell membrane in a lysine dependent manner, thereby opening the apo(a) structure to allow covalent Lp(a) formation. *In vivo* human kinetics models have provided evidence to support all possible scenarios for intracellular assembly, extracellular assembly and components of the process occurring in both locations, as well as use of apo(a) or apoB recycled from internalized Lp(a) [70–76]. Intriguingly, however, it has been reported using this approach that there exists a separate pool of apoB-100 intracellularly that binds to apo(a) to form Lp(a) particles, and that the rate of production of Lp(a)-apoB is different than that of either VLDL-apoB or LDL-apoB [70]. Our own evidence from a cultured hepatocyte model is consistent with this concept, where we found that apo(a) and apoB interact non-covalently within the cell, and that this interaction dictates the secretion rate of apo(a) [77].

## Removal of Lp(a) from the circulation

### Role of the liver in Lp(a) catabolism

Although steps in the production of Lp(a) are the major determinant of Lp(a) levels, a role for catabolism has been identified using *in vivo* human kinetics in which plasma Lp(a) levels are negatively correlated with the fractional catabolic rates (FCRs) of both apo(a) and apoB-100 components of Lp(a) [72]. In humans, the FCR of Lp(a) is ∼40% higher for LDL than for Lp(a) [78]. The liver is the primary site of Lp(a) catabolism, with apo(a) playing the major role in mediating Lp(a) particle uptake [79]. A number of receptors have been suggested to play a role in Lp(a) catabolism including the LDL receptor (LDLR; apoB/E receptor) and related family members including LRP-1 (LDLR-related protein-1) and the VLDL receptor, as well as SR-B1 (Scavenger Receptor Type B Class 1), megalin/gp330, the asialoglycoprotein receptor, and plasminogen receptors (Figure 3) [80,81].

The role of the LDLR in Lp(a) catabolism has been highly debated, with evidence both for, and against a role for this receptor [80]. *In vitro* data using different cell types have been relatively consistent in identifying high affinity binding and uptake of Lp(a) through the LDLR [82–85], although kindred studies from FH patients have reported conflicting data with respect to whether or not Lp(a) levels are elevated in patients with LDLR mutations [81]. Nonetheless, it had generally been accepted that Lp(a) is not an effective ligand for the LDLR in humans given that statins do not lower Lp(a) but may, in fact, slightly increase it [86]. It therefore came as a surprise when therapeutic antibody inhibitors of proprotein convertase subtilisin/kexin type 9 (PCSK9) lowered Lp(a) by 15–30% [87,88]. Romagnuolo et al. [83] subsequently showed that PCSK9 could reduce Lp(a) internalization by LDLR using cultured hepatoma cells and function blocking monoclonal antibodies against the LDLR; similar results were found using fibroblasts from FH patients versus healthy control and using primary liver hepatocytes from mice [83,84]. We also found that Lp(a) clearance was accelerated in PCSK9 knockout mice and delayed in LDLR knockout mice, with double-knockout animals having the same Lp(a) clearance rate as LDLR knockout mice (i.e. the defect in clearance could not be rescued by deletion of PCSK9) [89]. *In vivo* human kinetic studies were also performed to probe the basis for Lp(a)-lowering by PCSK9 inhibitors. Interesting, effects on Lp(a) production rate and/or FCR were observed, in some cases depending on whether patients were also receiving a statin [90–92]. It possible that Lp(a) may function as a ligand for the LDLR under specific conditions where Lp(a) levels are significantly elevated, LDL levels are low, and LDLR is substantially up-regulated by statins and PCSK9 inhibitors. This may explain results from catabolic studies using injected radiolabeled Lp(a) in mice overexpressing the LDLR; in these animals, Lp(a) clearance rates were dramatically increased, suggesting a role for the LDLR in Lp(a) uptake [93]. Importantly, however, mice also have very low levels of competing LDL, thereby favoring Lp(a) uptake through the LDLR. Nonetheless, the balance of the evidence generated to date is consistent with the idea that the LDLR is not the major clearance receptor for Lp(a) in liver.

The LRP (LDL related receptor) is a hepatic receptor in the LDL receptor (LDLR) family that mediates the hepatic uptake of chylomicrons and apoE-containing VLDL remnants. *In vitro* data suggest that LRP specifically may play a role in catabolism of high molecular mass Lp(a) isoforms [94]. However, this has not been confirmed in other studies [95,96]. It has also been reported that SR-BI can participate in Lp(a) catabolism: as is the case for both HDL and LDL, the SR-B1 receptor is involved in selective uptake of cholesteryl esters from the Lp(a) particle [97,98]. Additionally, it has been shown that the apo(a) and apoB-100 components of Lp(a) are can also internalized by this receptor [97]. Although the SR-B1 expression in liver is modest compared with adrenal glands and placenta, Remaley and colleagues have reported genetic variants of *SCARB1* that are associated with both elevated Lp(a) and HDL levels [98]. These include exonic variants with diminished SR-B1 activity that are associated with reduced Lp(a) binding and uptake.

The asialoglycoprotein receptor, present in high levels on the liver, is responsible for desialylated protein clearance. However, despite the extensive sialic acid modification of apo(a), there is no experimental evidence to suggest a role for this receptor in Lp(a) catabolism [79]. The megalin/gp330 receptor has been shown to function as a receptor for Lp(a) through the apoB100 moiety [99], but is not found in liver. However, this receptor is present in proximal tubules of the kidney which has been proposed as a site for Lp(a) catabolism [100].

Tam et al. [82] also identified a low-affinity, high-capacity receptor on hepatocytes which they suggested could correspond to members of the plasminogen receptor family. A role for the plasminogen receptor PLG-R_KT_ in Lp(a) catabolism has also been reported [101]. This receptor appears to participate in binding and internalization of Lp(a) based on *in vitro* studies in established hepatocyte cell lines. Although interesting, it is not clear what role this receptor plays *in vivo*, and whether other members of the plasminogen receptor family are also involved in Lp(a) catabolism. The PLG-R_KT_ receptor is expressed in many different tissues including brain, vasculature, and epithelial tissues such as lung and kidney and has a number of functions ascribed to it; the functions are predominantly centered on the role of this receptor in plasminogen activation to plasmin [97]. The potential ability for Lp(a) to compete with plasminogen to bind to this receptor clearly requires further study.

Most recently, a role for PLG-R_KT_, in addition to two other plasminogen receptors on liver cells (annexin A2, and S100A10 (S100 calcium-binding protein A10)) have been reported to facilitate the uptake of Lp(a) *in vitro* through the process of macropinocytosis [102]. Since these are not internalization receptors, the authors proposed that PLG-R_KT_ interacts with the apo(a) moiety of Lp(a) at the cell surface, followed by uptake of Lp(a) by macropinocytosis through the cell surface binding activity of the annexin A2 tetramer. Although potentially interesting, a number of questions remain. Hepatocytes are not known to perform macropinocytosis; this process is primarily associated with non-receptor mediated engulfment of cargo in immune cells [103]. Currently, no *in vivo* data have been presented to support either a physiological role for these plasminogen receptors in Lp(a) uptake, or the involvement of macropinocytosis in Lp(a) catabolism. Considering all the available evidence, we believe that the true Lp(a) receptor, if it exists at all, remains unidentified.

## Genetics of Lp(a)

### Regulation of Lp(a) levels by the *LPA* gene

Lp(a) is the most common monogenic cause of inherited CHD. In this regard, plasma Lp(a) concentrations are primarily determined at the level of the *LPA* gene with estimates of heritability of Lp(a) concentrations exceeding 90% [10]. Plasma Lp(a) concentrations reflect a co-dominant inheritance pattern where Lp(a) levels correspond to the sum of the contribution of each *LPA* allele. The majority of the variability in Lp(a) levels arises from the *LPA* gene itself [104]; this is primarily conferred by differences in *LPA* size where smaller alleles are strongly associated with higher plasma Lp(a) levels as well as by SNPs. Given the strong contribution of the *LPA* gene to plasma Lp(a) concentrations, levels are relatively resistant to lowering through alterations in diet and lifestyle or most lipid-lowering therapies [12].

Both median Lp(a) levels and the distribution of levels vary between different ancestry groups. It is known that Blacks have the highest Lp(a) level of all ancestry groups, followed by South Asians, European Caucasians, Hispanics, and East Asians [105]. Interestingly, the distribution of Lp(a) levels in the Black population are near-normal in their distribution, versus the positively skewed distribution in other ancestry groups. Lp(a) levels in European Caucasians are highly positively-skewed, while South Asians have a distribution intermediate to that observed in Whites and Blacks. Haplotype studies have shown that both *LPA* SNPs and KIV2 copy number are genomic determinants of plasma Lp(a) concentration, but that the extent of these contributions differs across ethnic groups [106]. SNPs that have been identified in *LPA* haplotypes are linked to both plasma Lp(a) levels and KIV2 copy number in Caucasian, Asian and South Asian populations. However, the KIV2 copy number explains a larger proportion of variability in plasma Lp(a) levels in European Caucasians, compared with that observed in the Asian or South Asian populations [106]. This may reflect the distribution of the rs10455872 SNP, which is only found in European Caucasians, and which is significantly associated with both KIV2 copy number and Lp(a) levels. Importantly, many SNPs are common in some ancestry groups but absent in others, indicating that they may play a role in the different relationships between *LPA* allele size and Lp(a) levels in these groups [11].

The KIV2 repeat region has been comparatively impenetrable to discovery of mutations because of its highly repetitive nature. However, advances in next-generation sequencing have allowed recent insights into the mutation burden in this region. Deep sequencing of this region has revealed the presence of many mutations (missense mutations as well as a substantial number of loss-of-function and splice-site mutations) that affect Lp(a) levels beyond that attributable solely to the number of KIV2-encoding sequences [11]. Several mutations resulting in ‘null’ *LPA* alleles have been reported: these can take the form of either nonsense codons or splice-site mutations [107,108]. Importantly, null alleles are strong predicters of both lower Lp(a) levels and reduced risk for CHD [107].

A strong implication of the finding of a very high mutation burden in the KIV2-encoding sequence is that *LPA* has characteristics of an expressed pseudogene. This, in turn, raises the question as to whether there is a critical physiological role for Lp(a), separate from its contributions to the pathophysiology of CVD. The remains highly debated since no physiological function(s) of Lp(a) to date have been confirmed.

### Relationship between *LPA* and other genes

GWAS has revealed three protein-encoding genes: *APOE*, *APOH* and *CETP*, outside the *LPA* gene cluster on chromosome 6, that are significantly associated with Lp(a) levels [104,109]. A 65% increase in Lp(a) mass was found to be associated with the apoE4-encoding ε4/ε4 genotype compared with apoE2-encoding ε2/ε2 genotype [110]. The ε2/ε2 genotype is associated with elevated triglycerides due to the impaired association of apoE2 with the LDLR; he basis of this association with Lp(a) levels remains unclear. The *APOH* locus encodes the protein beta2-glycoprotein I (β2GP1.) This plasma protein has a high affinity for negatively charged phospholipids. *In vitro*, β2GP1 has been reported to bind to Lp(a), both through the phospholipids on LDL as well as through the KIV domain of apo(a) [111]. However, the effect of these interactions on Lp(a) levels remains unclear. The identification of *CETP* in this GWAS analysis is consistent with the ability of CETP inhibitors to lower human Lp(a) levels in plasma [112,113]. However, the mechanism of action underlying these observations remains unclear and may reflect effects on either production or catabolism of Lp(a).

## Mechanism of action of Lp(a) in CVD

**Figure 4.**
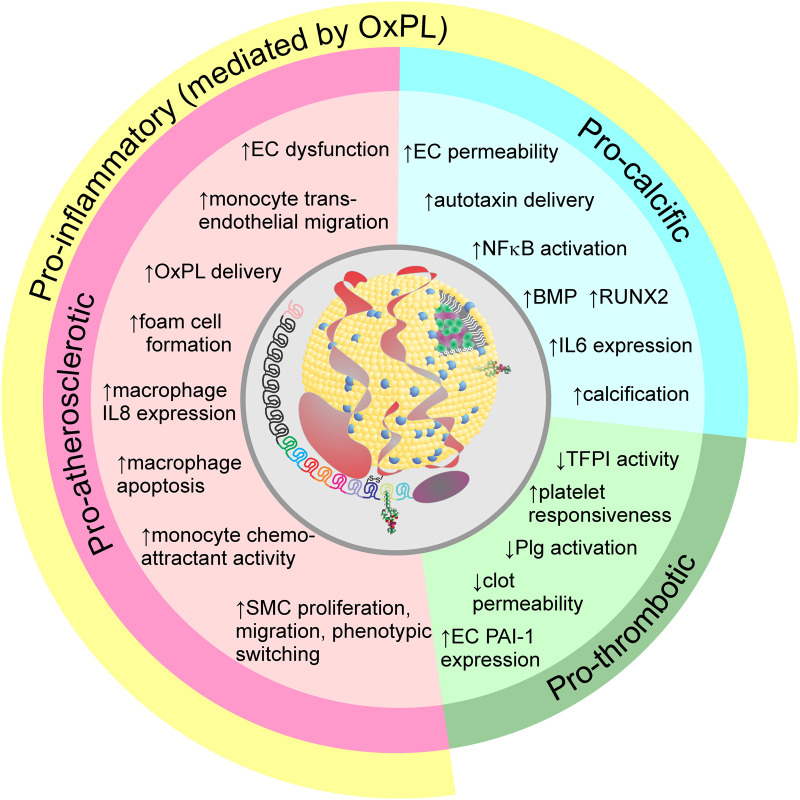
Potential pathogenic mechanisms of Lp(a). Lp(a) has pro-atherosclerotic, pro-calcific (primarily leading to calcific aortic valve disease), and pro-thrombotic mechanisms of action. Virtually all of the pro-atherosclerotic and pro-calcific effects have been shown to be mediated by the oxPL covalently associated with apo(a) KIV10. The individual pathogenic mechanisms have been primarily demonstrated through *in vitro* and *ex vivo* studies (see [115–117] for details). EC, endothelial cells; oxPL: oxidized phospholipids; IL: interleukin; SMC, smooth muscle cell; TFPI, tissue factor pathway inhibitor; Plg: plasminogen; PAI-1: plasminogen activator inhibitor type 1.

Based on Mendelian randomization studies, a causal relationship between elevated levels of Lp(a) and ASCVD or calcific aortic valve disease (CAVD) has been demonstrated [114]. However, the mechanism of action of Lp(a) at the cellular and molecular levels that underlies these observations remains unclear. This reflects the lack of an animal model that has the human *LPA* gene, making it suitable for manipulation in a disease model. As such, much of our mechanistic insights into Lp(a) pathogenicity have come from *in vitro* vascular cell studies (Figure 4). While transgenic apo(a)/Lp(a) mouse and rabbit models have been reported, they usually have significant limitations including Lp(a) levels below the pathogenic range, non-physiological truncated apo(a) variants, and the absence of human apoB100 to allow formation of a covalent Lp(a) particle in plasma [118]. Most recently, a novel transgenic mouse expressing a 13-kringle form of human apo(a) and human apoB100 has been reported that features very high plasma levels of bona fide human Lp(a) [119]. After 12 weeks of a high-fat high-cholesterol diet in the presence of antisense oligonucleotide-mediated *Ldlr* knockdown, female Lp(a) transgenic mice had higher aortic sinus plaque area, as well as increased calcification, oxidized phospholipid deposition, and necrotic core area compared with control mice expressing only human apoB100. The increased vulnerable plaque features seen in these animals will make them a useful model for understanding the role of Lp(a) in the initiation and progression of atherosclerosis.

### Determining a role for Lp(a) in thrombosis

When the cDNA sequence of apo(a) was published by McLean et al. [8], a striking similarity between apo(a) and the proteolytic precursor plasminogen was observed. When plasminogen is activated to plasmin by either tPA or uPA action, the resulting enzyme cleaves many substrates including fibrin, resulting in dissolution of thrombi through fibrinolysis. Based on its similarity with plasminogen, it was hypothesized that apo(a) and Lp(a) could inhibit this process. However, the data supporting a role for Lp(a) in fibrinolysis *in vivo* have been largely disappointing [115]. For example, Mendelian randomization studies suggested against a causal role of elevated Lp(a) in venous thromboembolism [120]. Although apo(a) alone is very effective at inhibiting fibrinolysis and plasminogen activation [121–123], the same does not appear to be true for the intact Lp(a) particle [124]. Indeed, in an *ex vivo* plasma clot lysis study using samples from patients in clinical trials of a potent Lp(a)-lowering antisense oligonucleotide, no effect of Lp(a) lowering on clot lysis time was observed [124]. However, this does not rule out a potential role for Lp(a) in the promotion of thrombus formation in the vasculature. For example, Lp(a) may modulate platelet reactivity and/or stimulate the pathways that result in thrombus formation and/or clot stabilization [115]. These mechanisms would operate independently of the similarity between apo(a) and plasminogen, pointing to a unique role for Lp(a) in ASCVD. In this regard, it has been shown that Lp(a) can inhibit tissue factor pathway inhibitor, thereby promoting coagulation [125]. Additionally, it has been reported that Lp(a) can increase PAI-1 expression in endothelial cells [126], and may play a role in promoting platelet aggregation based on *in vitro* findings [127]. In this regard, patients with elevated Lp(a) levels show greater benefit from prolonged dual antiplatelet therapy than patients with low Lp(a) levels [128]. In the ASPREE trial of aspirin in primary prevention in older adults, the benefit of this intervention was restricted to those subjects with a genetic signature of elevated Lp(a), without a significant increase in bleeding events [129]. Also consistent with a procoagulant role for Lp(a) are the results of a Mendelian randomization study which showed that elevated Lp(a) is in fact associated with a lower risk of major bleeding in the brain and airways [130].

### Mechanism of Lp(a) action in atherosclerosis and aortic valve disease — a role for Lp(a)-oxPL

In a series of studies involving ∼30 000 subjects [37,131–135], a very strong correlation between the oxPL/apoB ratio (measured using E06 and an apoB antibody in a plate assay) and Lp(a) concentrations was observed (*r* = 0.8–0.9) with the strength of the correlation inversely proportional to apo(a) isoform size [134]. Elevated oxPL/apoB levels have been shown to be associated with worse cardiovascular outcomes [136] and are positively associated with CAD as well as the progression of carotid, coronary and femoral atherosclerosis [133,134]. In the prospective 10-year follow up of the Bruneck study, oxPL/apoB was shown to strongly predict new CVD events and was independent of all known risk factors except for Lp(a) [137]. Importantly, levels of oxPL/apoB are predictive of new future CVD events beyond those predicted by the Framingham Risk Score [138]. Subsequent reports have linked the oxPL in Lp(a) to the pathogenesis of CAVD [139,140], which is notable since CAVD is not an atherothrombotic disease, despite sharing some risk factors [139]. These findings underscore the emerging concept that the effects on vascular cells of the apo(a) moiety contribute to the pathogenicity of Lp(a), while also serving to highlight oxPL as a mechanistic link between these observations.

The key observation that Lp(a), and not LDL, is the preferential carrier of oxPL in plasma has provided a different lens through which to consider the mechanistic basis of Lp(a) pathogenicity (Figure 4) [116]. OxPL species are highly proinflammatory, and their specific interaction with Lp(a) provides a potential avenue for delivery and retention of these species in the vessel at the site of atherosclerotic lesions. In addition of oxPL species present on the LDL component of Lp(a), apo(a) contains a covalently-linked oxPL moiety within its KIV10 domain, the addition of which requires the strong LBS present in this kringle [42]. The mechanism underlying the role of the LBS in mediating oxPL coordination is a subject of ongoing investigation.

Lp(a) accumulates and is retained in the site of developing atherosclerotic lesions to greater extent than LDL; the extent of accumulation is proportional to the plasma Lp(a) concentration [141]. The retention of Lp(a) in the intima is likely facilitated by its ability to bind to several extracellular matrix proteins including collagen, fibrinogen, and fibronectin. This provides a route by which Lp(a) can deliver oxPL to arterial lesions [142]. The oxPL present on apo(a) has been linked to a number of proinflammatory effects of Lp(a) including increased expression of inflammatory mediators in cultured macrophages and human monocytes [59,143]. The oxPL on apo(a) has also been implicated in alteration of vascular endothelial cell phenotype through activation of glycolysis, promoting adhesion and transmigration of monocytes across the endothelium [144]. This is in keeping with previous studies that have demonstrated a role for Lp(a) in endothelial cell signaling pathways that result in cellular contraction and disruption of cell-cell adhesion molecules, leading to endothelial dysfunction [145,146]. It has recently been shown that Lp(a), and to a lesser extent apo(a), increases caspase activation and release of IL-1B and IL-18 in human monocyte-derived macrophages and cultured THP-1 macrophages [7]. Interestingly, the oxPL moiety in apo(a) has also been shown to play a role in the development of CAVD through its ability to stimulate osteogenic differentiation in isolated valve interstitial cells [147]. As such, it has been hypothesized that OxPL-Lp(a) provides a link between ASCVD and CAVD in large part due to its proinflammatory properties [117].

The exact composition of the OxPL associated with apo(a) and the lipid portion of Lp(a) is not known. However, in addition to covalently-linked OxPL on apo(a), it is likely that OxPL associated with the lipid component of the Lp(a) particle may also contain oxidized adducts that contribute to proinflammatory responses. Although the lipidome of Lp(a) remains poorly characterized, it has been shown that it is enriched in diacylglycerols and lysophosphatidic acid (LPA), which contribute to proinflammatory responses in monocytes [43].

The mechanism by which Lp(a) contributes to phenotypic changes in aortic valve interstitial cells, vascular endothelial cells and monocytes/macrophages remains unclear with respect to initiation of signaling pathways that lead to programs of proatherosclerotic and procalcific gene expression in these cells. A role for Toll-like receptors has been suggested in monocytes and macrophages [116]. In VICs, oxPL bound to Lp(a) appears to be activated by phospholipase A2 to form lysophosphatidyl choline (LPC); autotaxin, also associated with Lp(a), then converts LPC to lyosphophatidic acid [140]. LPA, in turn binds to the LPA receptor on valve endothelial cells, initiating a cascade that leads to mineralization and osteogenic transition of valve interstitial cells, resulting in valve calcification [140].

Taken together, the role of Lp(a) in the progression of atherosclerosis is and CAVD is likely dependent in large part on its unique proinflammatory properties. This helps to explain the independent nature of the risk associated with Lp(a) and vascular diseases.

## Pharmaceutical approaches to Lp(a) lowering

It has been a major challenge to develop compounds that can specifically and effectively lower plasma Lp(a) levels. Most existing lipid-lowering medications do not lower Lp(a), or lower Lp(a) to a lesser extent than they do LDL-C [148]. As a consequence, it has not been possible thus far to demonstrate a specific CVD benefit from Lp(a) lowering.

In general, compounds that lower apoB secretion also lower plasma Lp(a) levels, but to a much lesser extent than LDL-C; this includes, for example, mipomersin [149] and lomitapide [150]. Although niacin lowers LDL and Lp(a), the effect on Lp(a) lowering is more effective for larger isoforms of apo(a) [151], which generally correspond to lower plasma levels. Amongst therapies that increase LDLR expression, statins do not lower Lp(a) (and may in fact slightly raise it [86]). PCSK9 inhibitors have an Lp(a) lowering effect, but much less than that observed for LDL-C [152].

A new approach to Lp(a) lowering has been ushered in with the use of RNA-directed therapies (Figure 3). These agents specifically, and very effectively, lower Lp(a) as they target the *LPA* mRNA encoding apo(a). The first of these agents was pelacarsen (formerly known as IONIS-APO(a)-L_Rx_ and TQJ230), an antisense oligonucleotide with chemically modified phosphate backbones and ribose moieties to increase its stability by decreasing nuclease digestion and to enhance its affinity for the target mRNA; pelacarsen also contains a triantennary GalNAc modification at the 5- end to facilitate specific uptake into hepatocytes via the asialoglycoprotein receptor [153]. Pelacarsen is able to achieve up to 70% reduction in Lp(a) levels with once-monthly dosing; in Phase II studies in subjects with highly elevated Lp(a), Pelacarsen was able to lower Lp(a) to below 50 mg/dl (125 nmol/l) in over 90% of subjects [154]. Pelacarsen is currently being tested in several Phase 3 trials, including Lp(a)HORIZON (NCT04023552), a randomized, double-blind, placebo-controlled cardiovascular outcomes trial. Subjects (*n* = 8323) with Lp(a) ≥ 70 mg/dl (175 nmol/l) and pre-existing ASCVD were randomized to receive 80 mg pelacarsen subcutaneously every 4 weeks or placebo, with the primary endpoint of time to expanded major adverse cardiovascular events (MACE; cardiovascular death, non-fatal MI, non-fatal stroke and urgent coronary re-vascularization requiring hospitalization) in patients with Lp(a) ≥ 70 mg/dl (175 nmol/l) or ≥90 mg/dl (225 nmol/l) Recruitment has been completed and the anticipated study reporting date is May 2025.

Several siRNA-based Lp(a) lowering agents are at various stages of clinical development. These agents are able to achieve an even more dramatic lowering of Lp(a) (>90%) with less frequent subcutaneous dosing [155]. Olpasiran is a GalNAc-modified siRNA that is currently being tested in the OCEAN(a) (NCT05581303) cardiovascular outcomes trial. Approximately 7000 patients with a history of ASCVD and Lp(a) ≥ 200 nmol/l were randomized to receive either 75 mg olpasiran subcutaneously every 12 weeks or placebo. The primary endpoint is time to CHD death, myocardial infarction, or urgent coronary revascularization, and the anticipated study completion date is December 2026.

Lepodisiran, another GalNAc-modified siRNA, is currently being tested in the ACCLAIM-Lp(a) (NCT06292013) randomized, placebo-controlled cardiovascular outcomes trial. Approximately 12 500 patients with a history of ASCVD or at high risk for ASCVD and Lp(a) ≥ 175 nmol/l will be enrolled, with the primary endpoint of time to first event in a composite MACE (cardiovascular death, nonfatal myocardial infarction, nonfatal stroke, and urgent coronary revascularization). Mean follow-up time will be 4.5 years, and the anticipated study completion date is in 2029.

A completely different approach to Lp(a) lowering has recently been reported, in which small molecules were developed to bind to KIV7 and KIV8 and thus inhibit the non-covalent first step of assembly of the Lp(a) particle (Figure 3) [156]. The ultimate product of this program, muvalaplin (LY3473329) is tripartite molecule with each arm able to bind tightly to the LBS in these kringles, affording an exceedingly potent ability to inhibit Lp(a) assembly *in vitro* (IC50 of 0.09 nmol/l). The crystal structure of muvalaplin with isolated KIV8 shows each arm bound to one KIV8 molecule, invoking the possibility of multivalent binding of muvalaplin to apo(a) to potentiate its Lp(a)-lowering ability [156]. In a Phase 1 ascending-dose study, muvalaplin lowered Lp(a) levels as early as 24 h after the first dose, and a maximum Lp(a) lowering of ∼65% was observed at doses >100 mg/day [157]. Importantly, as was the case for the RNA-directed therapies no safety or tolerability issues were noted in this study.

With the development of these effective Lp(a)-lowering therapies, we are at last at the cusp of answering the long-standing question of whether specific Lp(a) lowering can reduce risk of cardiovascular events. Given the prevalence of elevated Lp(a) in patients with existing ASCVD (10–15% of such patients would have Lp(a) levels high enough to be eligible for the cardiovascular outcomes trials), these agents could have a major impact on residual risk of ASCVD events in secondary prevention. It will take larger and longer studies to assess if Lp(a) lowering could also reduce risk in the primary prevention population; as we understand more about the mechanisms through which elevated Lp(a) causes ASCVD events, such studies will likely take on a greater urgency.

Although we are on the cusp of being able to effectively and specifically lower Lp(a), an ongoing question in the field is whether or not Lp(a) has a physiological function that might be affected by Lp(a) lowering. This has been a difficult question to address due to the lack of animal models that express endogenous Lp(a) containing all of the critical aspects of its structure that are found in humans. For example, KIV10 in Old World monkeys and apes lacks the strong LBS in KIV10 and consequently does not contain OxPL bound to this kringle. Many of these species also lack the KV domain, the function of which remains unclear. Based on its ability bind OxPL, early speculation suggested that human Lp(a) may play a protective role by functioning as a preferential carrier for plasma OxPL in plasma, thereby reducing the abundance of these inflammatory species on LDL [158]. It has also been proposed that Lp(a) may have evolved to prevent excessive bleeding through its prothrombotic properties [130,159]. Although population-based genetic analyses suggest that that lifelong exposure to low Lp(a) levels is linked to longevity [160], increased risk for new onset type 2 diabetes as well as metabolic dysfunction-associated liver disease in individuals with low Lp(a) levels (<3.8 nmol/l) has been reported [161]. However, these relationships may not be causal in nature and require further study. Clearly, there is much left to learn about Lp(a) structure and its mechanisms of action in both the vasculature and more broadly in the human body. Increased fundamental knowledge of Lp(a) will help to address the many remaining questions related to its role in human health and disease.

## Abbreviations

ASCVD: atherosclerotic cardiovascular disease
CAVD: calcific aortic valve disease
CHD: coronary heart disease
CVD: cardiovascular disease
FCR: fractional catabolic rate
KIV: kringle IV
LBS: lysine-binding site
LDL: low density lipoprotein
LPC: lysophosphatidyl choline
MACE: major adverse cardiovascular event
PCSK9: proprotein convertase subtilisin/kexin type 9
tPA: tissue-type plasminogen activator
